# Mind the Heart: Electrocardiography-gated cardiac computed tomography-angiography in acute ischaemic stroke—rationale and study design

**DOI:** 10.1177/2396987320962911

**Published:** 2020-10-11

**Authors:** Valeria Guglielmi, Leon A Rinkel, Nina-Suzanne Groeneveld, Nick HJ Lobé, S Matthijs Boekholdt, Berto J Bouma, Ludo FM Beenen, Henk A Marquering, Charles BLM Majoie, Yvo BWEM Roos, Adrienne van Randen, R Nils Planken, Jonathan M Coutinho

**Affiliations:** 1 Department of Neurology, Amsterdam UMC, University of Amsterdam, Amsterdam, The Netherlands; 2 Department of Radiology and Nuclear Medicine, Amsterdam UMC, University of Amsterdam, Amsterdam, The Netherlands; 3 Department of Cardiology, Amsterdam UMC, University of Amsterdam, Amsterdam, The Netherlands; 4 Department of Biomedical Engineering and Physics, Amsterdam UMC, University of Amsterdam, Amsterdam, The Netherlands

**Keywords:** Acute stroke, cardiac emboli, computed tomography angiography

## Abstract

**Rationale:**

About one-third of ischaemic strokes are caused by cardioembolism, and a substantial proportion of cryptogenic strokes likely also originate from the heart or aortic arch. Early determination of aetiology is important to optimise management. Computed Tomography-angiography of the heart is emerging as an alternative to echocardiography to detect cardio-aortic sources of embolism in stroke patients, but its diagnostic yield in acute ischaemic stroke has not been thoroughly assessed.

**Hypothesis:** We hypothesise that electrocardiography-gated computed tomography-angiography of the heart and aortic arch, acquired in the acute phase in patients with ischaemic stroke, has a higher diagnostic yield than transthoracic echocardiography as a first-line screening method for detection of cardio-aortic sources of embolism.

**Methods and design:**

Mind the Heart is a single-centre prospective observational cohort study. We will include consecutive adult patients with acute ischaemic stroke who are potentially eligible for reperfusion therapy. Patients undergo non-electrocardiography-gated computed tomography-angiography of the aortic arch, cervical and intracranial arteries, directly followed by prospective sequential electrocardiography-gated cardiac computed tomography-angiography. Routine work-up for cardioembolism including 12-leads electrocardiography, Holter electrocardiography and transthoracic echocardiography is performed as soon as possible. The primary endpoint is the proportion of patients with a predefined high-risk cardio-aortic source of embolism on computed tomography-angiography versus transthoracic echocardiography in patients who underwent both investigations. Based on an expected 5% additional yield of computed tomography-angiography, a sample size of 450 patients is required.

**Conclusions:**

The Mind the Heart study will generate a reliable estimate of the diagnostic yield of echocardiography-gated cardio-aortic computed tomography-angiography performed in the acute phase of ischaemic stroke.

## Introduction and rationale

It is estimated that up to one-third of ischaemic strokes are due to cardioembolism.^1,2^ A common cardioembolic cause of stroke is a thrombus in the left atrial appendage due to (paroxysmal) atrial fibrillation, while less frequent causes include left ventricular thrombus following recent myocardial infarction, endocarditis, prosthetic valve pannus or thrombus and cardiac tumours. Moreover, in a substantial proportion of patients with non-lacunar ischaemic stroke, routine diagnostic work-up fails to identify a cause, the so-called embolic strokes of undetermined source (ESUS).^2^ Cardio-aortic abnormalities with a medium risk of embolism, such as patent foramen ovale (PFO), left atrial appendage slow flow and atherosclerotic plaques in the aortic arch,^3,4^ are thought to be important causes of ESUS.

Establishing stroke aetiology is essential for optimising patient management including secondary stroke prevention. Furthermore, two recent trials have shown that treating all ESUS patients with direct oral anticoagulants (DOACs) instead of antiplatelets does not reduce the rate of recurrent stroke, which indicates that it is important to distinguish aetiology within the ESUS category and study targeted therapies.^5,6^

As diagnostic work-up for potential cardioembolism, current guidelines recommend 12-lead ECG and cardiac rhythm monitoring to search for atrial fibrillation and transthoracic and transesophageal echocardiography (TTE and TEE) to identify intra-cardiac thrombi and other structural sources of embolism.^7^ However, visualising cardiac thrombi with echocardiography is challenging and diagnostic yield of echocardiography for detection of high-risk cardiac sources of embolism is generally limited. A large systematic review on echocardiography in patients with ischaemic stroke and transient ischaemic attack (TIA) reported a variable prevalence of high-risk cardiac sources of embolism ranging from 0% to 9%.^8^ This variability was attributed to heterogeneity in study populations and methodological limitations of the included studies.

Furthermore, for logistical reasons (lack of time, limited availability of personnel and equipment and patient-related factors), echocardiography is often delayed until the subacute or chronic phase after stroke, at which time the source of the embolism may have disappeared.^9^ Finally, not all patients with stroke of undetermined aetiology receive echocardiography – especially TEE.^10,11^ This may be explained in part due to logistical reasons, and because TEE is invasive, may require sedation and carries a small risk of complications.^12^

Studies suggest that cardiac CT-angiography (CTA) may be a promising alternative to echocardiography for the detection of cardiac thrombi and other cardio-aortic sources of embolism.^13–16^ Now that CTA of the cervical and intracranial arteries is routinely done to determine eligibility for endovascular treatment (EVT), and additional radiation associated with cardiac CTA is low with modern CT scanners,^17^ cardiac and complete aortic arch CTA could relatively easily be included in the acute phase imaging protocol. This could result in faster diagnosis and improved treatment of cardioembolic stroke and reduce the number of patients with ESUS. Previous small studies have demonstrated heart–brain axis CTA in the acute phase of ischaemic stroke is feasible and has a promising diagnostic yield.^18–21^ However, large prospective studies comparing the diagnostic value of acute phase cardiac CTA versus echocardiography in ischaemic stroke patients have not been performed.

The hypothesis of the Mind the Heart study is that ECG-gated CTA of the heart and aortic arch in patients with acute ischaemic stroke has a higher diagnostic yield than TTE as a first-line screening method for the detection of cardio-aortic sources of embolism.

## Methods

### Study design, eligibility, consent

Mind the Heart is a prospective observational single-centre cohort study, performed at Amsterdam UMC, a comprehensive stroke centre in the Netherlands. We will include consecutive adult patients with acute ischaemic stroke who – at the time of imaging – are considered potentially eligible for reperfusion therapy (intravenous thrombolysis (IVT) or EVT; Table 1). Since May 2018, patients with (suspected) acute ischaemic stroke undergo ECG-gated cardiac CTA as part of the standard care diagnostic work-up in our hospital. The implementation of ECG-gated cardiac CTA was done gradually as CT technologists received training on the new protocol. Stroke diagnosis is established by a neurologist, based on clinical evaluation, imaging data and exclusion of other diagnoses. Patients with TIA are excluded from the study. The medical ethics committee of Amsterdam UMC, location AMC approved the study (2018_017#C2018275) and the study protocol was registered at the Dutch Central Commission for Human Research (CCMO; NL64139.018.18). Patients or their legal representatives are asked for written informed consent for the use of patient data for research purposes and follow-up.

**Table 1.** Inclusion criteria.

1. Age ≥18 years
2. Acute ischaemic stroke
3. Potentially eligible for reperfusion therapy (intravenous thrombolysis and endovascular treatment) at the time of imaging
4. ECG-gated cardiac CT-angiography performed as part of the initial diagnostic work-up in the acute phase of ischaemic stroke
5. Written informed consent from patient or representative

CT: computed tomography; ECG: electrocardiography.

### Study procedures

#### Image acquisition

**Figure 1.**
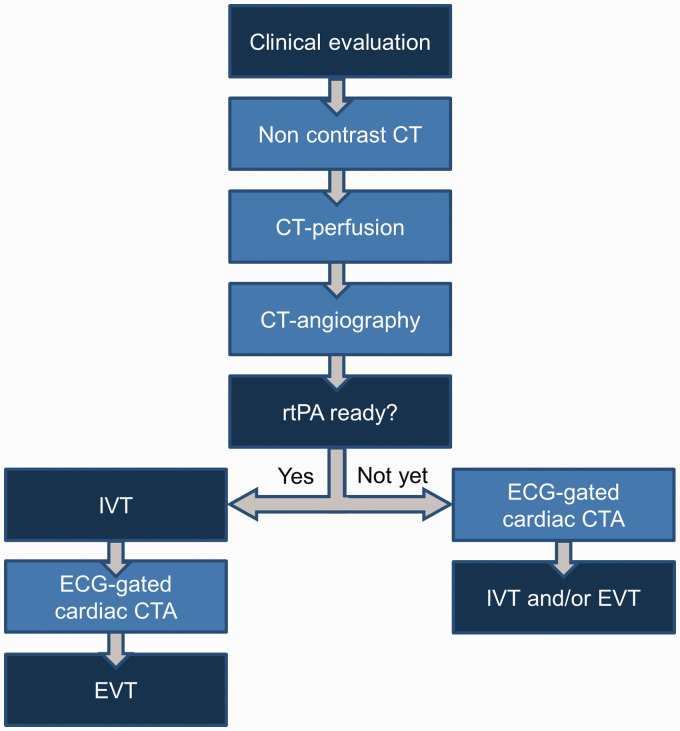
Code stroke practical workflow. Patients with suspected acute ischemic stroke are presented by the ambulance paramedics directly in the CT room. ECG-leads are placed prior to imaging. Non-contrast-enhanced CT of the brain, CT perfusion, non-gated CTA from the aortic arch to the intracerebral vessels are performed in supine, head first position with arms along body. Arms are then repositioned above head, followed by ECG-gated cardiac CTA, and IVT and/or EVT if indicated. If rtPA is ready for administration prior to cardiac CTA, IVT is administered prior to cardiac CTA. The patient receives IVT in the CT-room. CT: computed tomography; CTA: computed tomography-angiography; ECG: electrocardiography; EVT: endovascular treatment; IVT: intravenous thrombolysis; rtPA: recombinant tissue plasminogen activator.

Acquisition is done on a third-generation dual-source CT scanner (Somatom Force, Siemens Healthineers, Erlangen, Germany), which is located in the emergency department of the hospital. After a brief clinical examination in the CT scan room, patients sequentially undergo non-contrast-enhanced CT of the brain, CT perfusion, non-gated CTA of the aortic arch, cervical and intracranial arteries and prospective ECG-gated sequential cardiac CTA scan in diastole. Prospective ECG-gating entails that the scanner is triggered to scan the heart only during a predetermined phase of the cardiac cycle, as opposed to retrospective ECG-gating in which all phases are scanned and images of the phase of interest are selected retrospectively. For the code stroke practical workflow, see Figure 1. Technical details of the CTA protocol are available in Data Supplement I.

Acquisition times are recorded for each step of the imaging protocol. Radiation dose to the patient in Dose Length Product is registered and used to calculate the effective dose with the latest conversion guidelines.

Patients also undergo routine work-up for cardioembolism (ECG, Holter and TTE) in accordance with our national stroke guideline.^22^ Patients who do not require echocardiography as part of routine care undergo TTE as a study procedure, after informed consent. Patients <60 years undergo contrast TTE with agitated saline for the detection of PFO. TTE is performed as soon as possible, preferably within 72 hours of cardiac CTA. The decision to perform TEE in addition to TTE is at the discretion of the cardiologist.

#### Image interpretation

The on-call radiologist reports the images of the brain, extra- and intracranial arteries and screens for chest/cardiac findings that may directly impact patient management (e.g., acute aortic dissection, cardiac thrombus and pulmonary embolism). Subsequently, a comprehensive assessment of the heart, aortic arch and lungs is performed by a cardiac radiologist (RNP or AvR). Additional findings to the initial clinical report are communicated to the treating physician. TTE (and TEE, if performed) is evaluated by the on-call cardiologist, followed by a comprehensive assessment by cardiologist SMB or BJB. Similar to CTA, additional relevant findings to the initial clinical report are communicated to the treating physician. For both CTA and TTE, predefined structural sources of embolism from the heart and aortic arch (Data Supplements II and III) are systematically scored using a template. The cardiac radiologists and cardiologists who perform systematic scoring for study purposes are blinded to each other’s assessment.

#### Clinical data acquisition, patient follow-up and stroke aetiology

Detailed clinical information at baseline and follow-up are collected in a standardised manner using Castor Electronic Data Capture (EDC),^23^ an electronic case report form platform. At three month and two year follow-up, a structured interview by telephone is performed in order to determine functional outcome, expressed on the modified Rankin Scale. During the follow-up, we record secondary stroke prevention measures including medication and interventions, whether patients were treated for any cardio-aortic abnormalities, and whether a recurrent stroke or TIA has occurred. A comprehensive assessment of the carotid arteries, including evaluation for the presence of non-stenotic plaques^24,25^ and carotid artery webs,^26^ is performed by a neuroradiologist. An adjudication committee consisting of a neurologist, neuroradiologist, cardiac radiologist and cardiologist determine stroke aetiology according to the Trial of Org 10172 in Acute Stroke Treatment (TOAST) criteria,^27^ based on all acquired information. ESUS is diagnosed according to published criteria.^2^

### Outcomes

The primary outcome is the proportion of patients in whom a predefined high-risk cardio-aortic source of embolism is established with CTA, compared to TTE, in patients who underwent both investigations. High-risk cardio-aortic sources of embolism have been chosen based on available literature^2^,^27–29^ and consensus within the steering committee and are: left atrial appendage thrombus, left atrium thrombus, left ventricular thrombus, prosthetic valve abnormalities (pannus or thrombus), signs of endocarditis (e.g., valvular vegetations), severely enlarged left ventricle, atrial myxoma, papillary fibroelastoma, recent myocardial infarction, sings of rheumatic valvular disease (mitral stenosis), >4 mm ulcerated noncalcified (soft and mixed) aortic arch atherosclerotic plaques and Stanford Classification Type A acute aortic dissection. For detailed definitions of each cardio-aortic source on CTA and TTE, see Data Supplements II (high-risk) and III (medium/low-risk).

Secondary outcomes are: (1) comparison of two diagnostic strategies (CTA vs TTE), expressed as proportion of patients in which a high-risk cardio-aortic source is established with CTA, compared to TTE. Unlike in the primary analysis, in this analysis all enrolled patients are included, i.e., also those in whom TTE was not performed for whatever reason. This analysis provides insight into the diagnostic yield of each imaging technique in daily clinical practice; (2) inter-observer variability of cardio-aortic CTA reading; (3) impact of cardiac CTA findings and TTE findings on patient management, defined as change in medical management (anticoagulation, antibiotics and heart failure therapy) or any surgical or endovascular procedures in relation to the cardio-aortic finding; (4) differences between women and men in terms of prevalence, subtype, treatment and outcome of cardio-aortic causes of acute ischaemic stroke.

### Sample size

The sample size calculation is based on the additional diagnostic yield of CTA compared to TTE for high-risk cardio-aortic causes of acute ischaemic stroke, using assumptions that are based on a combination of preliminary data from our hospital and available literature.^14^ A sample size of 337 patients, each with CTA and TTE data, will achieve 80% power to detect an additional diagnostic yield of 5%, using a two-sided McNemar test with binomial enumeration with a significance level of 0.05 (Exact Sign test of Equality of Paired Proportions in nQuery Sample Size Software version 8.4.1.0, Statsols). Such an additional diagnostic yield occurs if the proportion of all patients, for whom TTE does not identify cardio-aortic causes, but CTA does, is 0.075. And if the proportion of all patients, for whom CTA does not identify cardio-aortic causes, but TTE does, is 0.025. Hence, the total proportion of all patients with discordant TTE and CTA results is 0.100. Assuming that 75% of included patients undergo both CTA and TTE, we chose a total sample size of 450 patients.

### Statistical analyses

To determine the diagnostic yield of CTA and TTE for detection of high-risk cardio-aortic causes of stroke, data of patients who underwent both CTA and TTE will be analysed by using a two-sided McNemar test with binomial enumeration (Mid-P Exact) and a significance level of 0.05.

For the comparison of two diagnostic strategies (secondary outcome 1), we will impute missing data using multiple imputation based on relevant covariates and outcome and then perform conditional logistic regression.

Inter-observer variability for CTA findings (secondary outcome 2) will be determined by calculating Cohen’s Kappa. Two cardiac radiologists independently evaluate high-risk cardio-aortic sources of embolism in a proportion of patients included in the study. Concordance between readers is defined as agreement on the presence or absence of all high-risk sources of embolism in that particular patient. In addition, we will score agreement between readers for each separate high-risk cardio-aortic cause of embolism. Assuming a minimum Kappa of 0.60, and an anticipated Kappa of 0.80, a sample of 52 patients is required if the prevalence of high-risk sources of embolism is 30%.^30^ In order to achieve the 30% prevalence rate, we will select a stratified sample with a relative overrepresentation of patients with cardio-aortic abnormalities compared to the study population.

To study the impact of cardiac CTA findings on patient management (secondary outcome 3), patients who received treatment (change in medical management, surgery or endovascular intervention) for cardio-aortic abnormalities on CTA will be descriptively reported, including the time from stroke diagnosis and establishment of aetiology to treatment.

To determine sex differences (secondary outcome 4), a stratified analysis for sex will be performed including stratification per age-group. Baseline characteristics will be summarised using descriptive statistics. Two-sided independent T-test will be used for comparison of continuous data, Chi-Square test or Fisher’s exact test for comparison of proportions, whichever is appropriate.

Statistical analyses will be performed using IBM SPSS Statistics for Windows, version 24.0 (Chicago, IL).

### Study launch and enrolment

The first patient was enrolled in the Mind the Heart study on 15 May 2018. At the time of submission (30 June 2020), 358 patients had been enrolled.

## Discussion

The overarching aim of the Mind the Heart study is to determine the diagnostic yield of ECG-gated cardiac CTA, performed in the acute phase work-up of ischaemic stroke, compared to TTE. If the data indicate that cardiac CTA has a similar or higher diagnostic yield than TTE, cardiac CTA may become an alternative to echocardiography to diagnose cardio-aortic sources of embolism in stroke patients. Moreover, since cardiac CTA can be performed relatively easily in the acute stroke diagnostic work-up, it could lead to faster diagnosis and improved treatment of cardioembolic stroke and reduce the number of patients who are diagnosed with ESUS.

In the Mind the Heart study, prospective ECG-gated cardiac CTA is performed instead of non-gated or retrospective ECG-gated cardiac CTA. The major advantage of prospective ECG-gated cardiac CTA is that it produces better quality images of the moving cardiac chambers and structures than non-gated CTA and requires less radiation than retrospective ECG-gated CTA.^31^ In contrast, the main advantage of non-gated cardiac CTA is fast acquisition, which makes it easier to implement in an acute ischaemic stroke setting. The largest study on acute phase non-gated CTA of the heart–brain axis thus far found high-risk cardio-aortic causes of ischaemic stroke in 15% of patients, but also reported poor cardiac image quality in 40% of patients.^20^ Higher image quality improves assessment reliability and likely increases diagnostic yield. The benefit of retrospective ECG-gated cardiac CTA is cardiac chamber visualisation throughout all phases of the cardiac cycle, which may increase the chance to acquire high-quality images in patients with atrial fibrillation or other heart rhythm irregularities and provides richer data on cardiac function. To the best of our knowledge, there is only one study of acute phase retrospective ECG-gated cardiac CTA in ischaemic stroke patients,^21^ but this study did not include data on diagnostic yield.

We chose TTE as comparator instead of TEE because TTE is recommended as the first-line screening method for imaging of the heart in ischaemic stroke patients with suspected cardioembolic source^12^ and is part of the standard stroke work-up prior to classification as ESUS.^2^ TEE is infrequently performed in ischaemic stroke patients, and generally only in selected patients and after TTE.^11^ The Mind the Heart study aims to compare the yield of CTA with echocardiography as performed in routine clinical care, and as such, using TTE as a comparator is more representative. Ideally, for a comparison of diagnostic yield, TTE and cardiac CTA would be performed at the same time. However, performing TTE as part of the initial acute phase work-up is not feasible without causing significant delay in reperfusion therapy. Doing so for the purpose of the study would not reflect clinical practice and would decrease the quality of care. Still, in order to minimise the time difference between the two diagnostic tests, we strive to perform TTE within 72 hours of CTA. Performing TTE at this time is quicker than typically reported in other studies in stroke patients.^5,16,32^

As our primary outcome we chose the diagnostic yield of cardiac CTA versus TTE, and not diagnostic accuracy of CTA versus TTE. We did so because we believe that due to the lack of a true gold standard for many cardio-aortic sources of embolism, presenting results in terms of sensitivity/specificity does not necessarily reflect the truth.^16^ Especially in cases in which TTE is more likely to present false-negative results, diagnostic accuracy does not do justice to the potential value of CTA.^16^ Diagnostic yield takes into account certain unique, non-overlapping qualities of both CTA and TTE.

If acute phase ECG-gated cardiac CTA has a higher diagnostic yield than TTE as a first-line screening method for detection of cardio-aortic sources of embolism, an important follow-up question would be what the influence of cardio-aortic CTA findings are on patient management. For certain findings such as endocarditis and aortic dissection, acute phase cardiac CTA likely increases the chance of correct diagnosis and management.^33^ However, evidence-based targeted management strategies are not yet available for stroke patients with other cardio-aortic findings such as large (ulcerated) plaques in the aortic arch.^34^ The optimal acute phase management of stroke patients with cardiac thrombi is also unclear. Studies suggest that early initiation of DOACs prevent early recurrences of embolic stroke due to atrial fibrillation, with low frequency of symptomatic intracerebral haemorrhage.^35^ Applying cardiac CTA in the acute phase may help stratify which patients benefit from early initiation, such as patients with large cardiac thrombi, but this requires further study. Finally, acute phase cardiac CTA could potentially help guide strategy for EVT, because it may provide insight into thrombus characteristics.^36,37^

The data of our study may help to contribute to the implementation of cardiac CTA as a routine technique in the diagnostic work-up of stroke patients. However, several challenges that may complicate this process of implementation should be taken into account. First, extending the CTA to include the heart while maintaining optimal door-to-needle (IVT) and door-to-groin times (EVT). Other main challenges will include the availability of ECG-gating hardware and software, remaining within patient tolerable ranges for contrast media volume, timely reporting of cardiac CTA findings by experienced radiologists and dealing with incidental findings.

### Limitations

As described, the lack of a true gold standard for many cardio-aortic sources of embolism is a limitation. Furthermore, the current study has a pragmatic observational cohort design in which patients undergo both acute phase cardiac CTA and TTE. An alternative design would have been a randomised trial where patients undergo acute phase cardiac CTA + TTE or TTE only. However, this design would require considerably more patients and would have logistical issues. A randomised trial design would require informed consent prior to CTA, which would delay acute treatment. Consequently, our study is not designed to conclusively evaluate the impact of cardiac CTA on patient management. Furthermore, the external validity of our single-centre study may be limited by the availability of similar CT scanners in other hospitals. Lastly, our study is not powered to detect sex differences in subtype prevalence, treatment and outcome in patients with cardio-aortic findings.

## Conclusions

The Mind the Heart study will provide a reliable estimate of the diagnostic yield of ECG-gated CTA of the heart and aortic arch acquired in the acute phase of ischaemic stroke. In time, cardiac CTA may replace echocardiography as the first-line screening method to detect sources of cardioembolism in these patients.

## Supplemental Material

sj-pdf-1-eso-10.1177_2396987320962911 - Supplemental material for Mind the Heart: Electrocardiography-gated cardiac computed tomography-angiography in acute ischaemic stroke—rationale and study design Click here for additional data file. Supplemental material, sj-pdf-1-eso-10.1177_2396987320962911 for Mind the Heart: Electrocardiography-gated cardiac computed tomography-angiography in acute ischaemic stroke—rationale and study design by Valeria Guglielmi, Leon A Rinkel, Nina-Suzanne Groeneveld, Nick HJ Lobé, S Matthijs Boekholdt, Berto J Bouma, Ludo FM Beenen, Henk A Marquering, Charles BLM Majoie, Yvo BWEM Roos, Adrienne van Randen, R Nils Planken and Jonathan M Coutinho in European Stroke Journal
