# Supplementary material for: Mind the Heart: Electrocardiography-gated cardiac computed tomography-angiography in acute ischaemic stroke—rationale and study design
Source: Eur Stroke J. 2020 Oct 11;5(4):441–8. doi: 10.1177/2396987320962911 (PMC7856589; doi:10.1177/2396987320962911)
Supplement: sj-pdf-1-eso-10.1177_2396987320962911 - Supplemental material for Mind the Heart: Electrocardiography-gated cardiac computed tomography-angiography in acute ischaemic stroke—rationale and study design [file sj-pdf-1-eso-10.1177_2396987320962911.pdf]

SUPPLEMENTARY MATERIAL

Mind the Heart: ECG-gated cardiac CT-angiography in acute ischemic stroke – rationale and study design

European Stroke Journal

Valeria Guglielmi, MD, Leon A. Rinkel, MD, Nina-Suzanne Groeneveld, Bsc, Nick H.J. Lobé, Bsc, S. Matthijs Boekholdt, MD, PhD, Berto J. Bouma, MD, PhD, Ludo F.M. Beenen, MD, Henk A. Marquering, PhD, Charles B.L.M. Majoie, MD, PhD, Yvo B.W.E.M. Roos, MD, PhD, Adrienne van Randen, MD, PhD, R. Nils Planken, MD, PhD, Jonathan M. Coutinho, MD, PhD

Correspondence to: Jonathan M. Coutinho, Department of Neurology, Meibergdreef 9, 1105 AZ Amsterdam, The Netherlands. E-mail: j.coutinho@amsterdamumc.nl, Tel: +31-20-5662004, ORCID: 0000-0002-8284-982X

Data Supplement I. Technical details of acute phase heart-brain axis CT-angiography (CTA) for ischemic stroke patients

**Protocol**	**CTA aortic arch – cerebral vessels**	**ECG-gated cardiac CTA**
Patient position	Supine, head first, arms along body	Supine, head first, arms above head
Scan direction	Caudo-cranial	Cranio-caudal
Tube voltage, ref. Kvp	100	100
Tube current, ref. mAs	200	288
Pitch	1.0	Not applicable
Rotation time, seconds	0.25	0.25
Slice thickness, mm	0.6	0.6
Vascular reconstructions: slice thickness and kernel	1.0 (increment 0.7 mm), Bv44	0.6 (increment 0.4 mm), Bv40
Contrast media volume, mL*	50 (300mgI/ml)	40 (300mgI/ml)
ECG-gating	No	Yes, prospective, set to scan during diastole (63-76% of the R-R interval)
Beta-blockers	No	No

*Total contrast media volume for acute phase imaging diagnostic work-up of ischemic stroke is 125mL (300mgI/ml), including 35mL for CT-perfusion. Contrast is injected at 6.0 mL/s into the right cubital vein with an 18-gauge intravenous catheter, followed by 40mL saline solution injected at 6.0 mL/s. For additional information feel free to contact the authors.

Data Supplement II. High-risk[1-4] structural cardio-aortic sources of ischemic stroke: definitions on CT-angiography (CTA) and transthoracic echocardiography (TTE)

	CTA	TTE
**Heart**		
Left atrial appendage thrombus	A filling defect that appears as a low-attenuated mass (typically <100 HU) in left atrial appendage.[5]	A circumscribed echogenic or echolucent mass in left atrial appendage, distinct from the surrounding atrial wall.[6]
Left atrial thrombus	A filling defect that appears as a low-attenuated mass (typically <100 HU) in left atrium.[5]	A circumscribed echogenic or echolucent mass in left atrium, distinct from the surrounding atrial wall.[6]
Left ventricular thrombus	A filling defect that appears as a low-attenuated mass (typically <100 HU) in the left ventricle, often attached to infarcted myocardial wall.[7, 8] Often concomitant with apical aneurysm.	A circumscribed echogenic or echolucent mass in the left ventricle, distinct from the surrounding ventricular wall.[6]
Recent myocardial infarction (<4 weeks)*	Hypodense myocardium in a coronary artery territory without evidence of myocardial thinning.[7]	Regional wall motion abnormalities with preservation of normal wall thickness and normal reflectivity.[9]
Severely enlarged left ventricle	Left ventricle diastolic volume. Severely enlarged, indexed for BSA is defined as >94 mL/m^2 for men and >86 mL/m^2 for women.[10†]	Left ventricle end diastolic volume, 2d echocardiography volume calculations according to the biplane method of disks summation (modified Simpson's rule).[11] Severely enlarged, indexed for BSA is defined as >100 mL/m^2 for men and >80 mL/m^2 for women.[11]
Signs of endocarditis	Suggestive of endocarditis[7]:<br>- Valve vegetation, perforation or thickening.<br>- Paravalvular abscess, paravalvular pseudoaneurysn, or infiltration of perivalvular fat tissue.	Suggestive of endocarditis[7]:<br>- Valve vegetation, perforation or thickening.<br>- Paravalvular abscess, paravalcvular pseudoaneurysm,<br>- Paravalvular leakage (prosthetic valves).
Prosthetic valve pannus or thrombus (bioprosthetic or mechanical)	Pannus underneath valvular apparatus or thrombus[7]	Pannus underneath valvular apparatus or thrombus.[7]
Myxoma	A large, round, low attenuating mass with a small stalk, often attached to the fossa ovale.[12]	A large, round mass with a small stalk, often attached to the fossa ovale.
Papillary fibroelastoma	A small mass, hypodense with irregular borders attached by a thin stalk, often on the aortic and mitral valves.[12]	A small mass with irregular borders attached by a thin stalk, often on the aortic and mitral valves.
Signs of rheumatic valvular disease (mitral stenosis)	Calcification on mitral valve or subvalvular apparatus (not mitral valve annulus).	≥Moderate mitral stenosis, mean gradient ≥5mmHg, valve area <1.5 cm².[13]
**Aortic arch**		
>4 mm ulcerated noncalcified (soft and mixed composition) atherosclerotic plaque	Measurement definitions[3]:<br>- <u>Size:</u> largest lesion wall thickness measured endoluminal – adventitia.<br>- <u>Ulceration:</u> crater >2mm in depth and width.<br>- <u>Composition</u>: calcified (high attenuation), mixed, soft (low attenuation).	N/A
Stanford classification type A acute aortic dissection	A flap and false lumen in the ascending aorta.[14]	A flap and false lumen in the ascending aorta.[14]

HU, Hounsfield units; N/A, not assessable; M, men; W, women.
[*] Estimate of timing based in part on patient history.
[†] Guidelines for quantification of left ventricle size are not available for CTA. As a cut-off for severely enlarged we chose available guidelines for cardiovascular magnetic resonance imaging.

Data Supplement III. Other (medium/low-risk[1-4]) structural cardio-aortic sources of ischemic stroke: definitions on CT-angiography (CTA) and transthoracic echocardiography (TTE)

	CTA	TTE
**Heart**		
Non chicken wing left atrial appendage morphology	Left atrial appendage morphology[15, 16]:<br>- Chicken wing: only one lobe, total length more than 4 cm, and a bend angle <100°.<br>- Cactus: dominant central lobe, one or more secondary lobes, total length <4 cm.<br>- Windsock: one dominant lobe with several secondary, or even tertiary lobes, total length >4 cm, and a bend angle >100°.<br>- Cauliflower: total length <4 cm and complex internal structures.	N/A
Left atrial appendage slow flow*	A filling defect that appears as a mass with a higher attenuation (typically >100 HU) in left atrial appendage.[5]	N/A
Severely enlarged left atrium	Left atrium diastolic volume. Severely enlarged volume indexed for BSA is defined as >48 mL/m^2 for men and women.[11†]	Left atrium endsystolic measurements in 2DE volume biplane calculations according to biplane disk summation technique measured from both the apical four and two chamber views.[11]<br>Severely enlarged volume indexed for BSA is defined as >48 mL/m^2 for men and women.[11]
Atrial diverticulum	Outpouch of left atrium wall with smooth contour, most commonly located in the right anterior-superior wall of the left atrium.[17]	Outpouch of left atrium wall with smooth contour, contraction synchronized with the rest of the heart.
Left ventricular apical aneurysm	Distinct area of abnormal left ventricular diastolic contour with wall thinning.[18]	Dyskinetic (presence of paradoxical wall motion) out-pouching area or zone of the left ventricle with wall thinning.[18]
Older myocardial infarction (>4 weeks, <6 months)[‡]	Hypodense myocardium in a coronary artery territory with myocardial thinning.[7, 8]	Regional wall motion abnormalities with a thin akinetic reflective segment.[9]
Left ventricular non-compaction cardiomyopathy	End diastolic ratio of non-compacted myocardium to compacted myocardium must be greater than 2.3 during the diastole.[19]	End systolic ratio of non-compacted to compacted myocardium above 2.[20]
Calcific aortic valve	Leaflet thickening and calcifications of aortic valve.[21]	Leaflet thickening and calcifications of aortic valve.[21]
Patent foramen ovale	A crypt shaped contrast jet from the left atrium to the right atrium towards the vena cava, or an atrium septum discontinuity.[8, 22]	Rapid intravenous injection of agitated saline opacifies the right heart with microbubbles. The appearance of microbubbles in the left atrium within 3–6 cardiac beats after opacification of the right atrium indicates a patent foramen ovale.[23] Right-to-left shunt may be accentuated by the Valsalva manoeuvre.
Atrial septal defect	A contrast jet from the left atrium to the right atrium, or an atrium septum discontinuity.[8]	An atrium septum discontinuity with a color doppler jet from the left atrium to the right atrium.[23]
Ventricular septal defect	A contrast jet from the left ventricle to the right ventricle, or a ventricular septum discontinuity.[8]	A ventricular septum discontinuity with a color doppler jet from the left ventricle to the right ventricle.[24]
**Aortic arch**		
Atherosclerotic plaque	Plaque in any of these locations: aortic root, ascending aorta, aortic arch, descending aorta.	N/A

HU, Hounsfield units; N/A, not assessable; M, men; W, women.

* Other terms for the same phenomenon: left atrial turbulence (smoke), circulatory stasis, reduced flow velocities, spontaneous echodensities.

† Guidelines for quantification of left atrial size are not available for CTA. As a cut-off for severely enlarged we chose available guidelines for echocardiography.

‡ Estimate of timing based in part on patient history.

References Data Supplements II and III

1. Adams HP, Jr., Bendixen BH, Kappelle LJ, et al. Classification of subtype of acute ischemic stroke. Definitions for use in a multicenter clinical trial. TOAST. Trial of Org 10172 in Acute Stroke Treatment. *Stroke* 1993; 24: 35-41.
2. Hart RG, Diener HC, Coutts SB, et al. Embolic strokes of undetermined source: the case for a new clinical construct. *Lancet Neurol* 2014; 13: 429-438.
3. Kamel H and Healey JS. Cardioembolic Stroke. Circ Res 2017;120:514-526.
4. Yang H, Nassif M, Khairy P, et al. Cardiac diagnostic work-up of ischaemic stroke. *Eur Heart J* 2018; 39: 1851-1860.
5. Teunissen C, Habets J, Velthuis BK, et al. Double-contrast, single-phase computed tomography angiography for ruling out left atrial appendage thrombus prior to atrial fibrillation ablation. *Int J Cardiovasc Imaging* 2017; 33: 121-128.
6. Ko SB, Choi SI, Chun EJ, et al. Role of cardiac multidetector computed tomography in acute ischemic stroke: a preliminary report. *Cerebrovasc Dis* 2010; 29: 313-320.
7. Pagan RJ, Parikh PP, Mergo PJ, et al. Emerging role of cardiovascular CT and MRI in the evaluation of stroke. *AJR Am J Roentgenol* 2015; 204: 269-280.
8. Kamalian S, Kamalian S, Pomerantz SR, et al. Role of cardiac and extracranial vascular CT in the evaluation/management of cerebral ischemia and stroke. *Emerg Radiol* 2013; 20: 417-428.
9. American College of Cardiology Foundation Appropriate Use Criteria Task F, American Society of E, American Heart A, et al. ACCF/ASE/AHA/ASNC/HFSA/HRS/SCAI/SCCM/SCCT/SCMR 2011 Appropriate Use Criteria for Echocardiography. A Report of the American College of Cardiology Foundation Appropriate Use Criteria Task Force, American Society of Echocardiography, American Heart Association, American Society of Nuclear Cardiology, Heart Failure Society of America, Heart Rhythm Society, Society for Cardiovascular Angiography and Interventions, Society of Critical Care Medicine, Society of Cardiovascular Computed Tomography, Society for Cardiovascular Magnetic Resonance American College of Chest Physicians. *J Am Soc Echocardiogr* 2011; 24: 229-267.
10. Kawel-Boehm N, Maceira A, Valsangiacomo-Buechel ER, et al. Normal values for cardiovascular magnetic resonance in adults and children. *J Cardiovasc Magn Reson* 2015; 17: 29.
11. Lang RM, Badano LP, Mor-Avi V, et al. Recommendations for cardiac chamber quantification by echocardiography in adults: an update from the American Society of Echocardiography and the European Association of Cardiovascular Imaging. *Eur Heart J Cardiovasc Imaging* 2015; 16: 233-270.
12. Kassop D, Donovan MS, Cheezum MK, et al. Cardiac Masses on Cardiac CT: A Review. Curr Cardiovasc Imaging Rep 2014;7:9281.
13. Baumgartner H, Falk V, Bax JJ, et al. 2017 ESC/EACTS Guidelines for the management of valvular heart disease. *Eur Heart J* 2017; 38: 2739-2791.
14. Fukui T. Management of acute aortic dissection and thoracic aortic rupture. *J Intensive Care* 2018; 6: 15.

15. Wang Y, Di Biase L, Horton RP, et al. Left atrial appendage studied by computed tomography to help planning for appendage closure device placement. *J Cardiovasc Electrophysiol* 2010; 21: 973-982.
16. Korhonen M, Muuronen A, Arponen O, et al. Left atrial appendage morphology in patients with suspected cardiogenic stroke without known atrial fibrillation. *PLoS One* 2015; 10: e0118822.
17. Holda MK, Koziej M, Wszolek K, et al. Left atrial accessory appendages, diverticula, and left-sided septal pouch in multi-slice computed tomography. Association with atrial fibrillation and cerebrovascular accidents. *Int J Cardiol* 2017; 244: 163-168.
18. Lapeyre AC, 3rd, Steele PM, Kazmier FJ, et al. Systemic embolism in chronic left ventricular aneurysm: incidence and the role of anticoagulation. *J Am Coll Cardiol* 1985; 6: 534-538.
19. Petersen SE, Selvanayagam JB, Wiesmann F, et al. Left ventricular non-compaction: insights from cardiovascular magnetic resonance imaging. *J Am Coll Cardiol* 2005; 46: 101-105.
20. Jenni R, Oechslin E, Schneider J, et al. Echocardiographic and pathoanatomical characteristics of isolated left ventricular non-compaction: a step towards classification as a distinct cardiomyopathy. *Heart* 2001; 86: 666-671.
21. Khetarpal V, Mahajan N, Madhavan R, et al. Calcific aortic valve and spontaneous embolic stroke: a review of literature. *J Neurol Sci* 2009; 287: 32-35.
22. Pathan F, Hecht H, Narula J, et al. Roles of Transesophageal Echocardiography and Cardiac Computed Tomography for Evaluation of Left Atrial Thrombus and Associated Pathology: A Review and Critical Analysis. *JACC Cardiovasc Imaging* 2018; 11: 616-627.
23. Silvestry FE, Cohen MS, Armsby LB, et al. Guidelines for the Echocardiographic Assessment of Atrial Septal Defect and Patent Foramen Ovale: From the American Society of Echocardiography and Society for Cardiac Angiography and Interventions. *J Am Soc Echocardiogr* 2015; 28: 910-958.
24. Penny DJ and Vick GW, 3rd. Ventricular septal defect. *Lancet* 2011; 377: 1103-1112.
